# Mapping the evidence of hepatoprotective properties of *Moringa oleifera* from sub-Saharan African countries: a systematic review protocol

**DOI:** 10.1186/s13643-019-1117-2

**Published:** 2019-08-08

**Authors:** Willy Tambwe Muzumbukilwa, Mukanda Gedeon Kadima, Manimbulu Nlooto, Peter Mark Oroma Owira

**Affiliations:** The Discipline of Pharmaceutical Sciences, School of Health Sciences, Westville Campus, University of KwaZulu-Natal, University Road, Durban, 4001 South Africa

**Keywords:** *Moringa oleifera*, Alanine aminotransferase, Aspartate aminotransferase, Gamma-glutamyltransferase, Alkaline phosphatase, Superoxide dismutase, Catalase, Glutathione reductase, Hepatoprotective, Sub-Saharan Africa countries

## Abstract

**Background:**

One of the most challenging health problems is liver disease, which can be caused by medications, toxic substances, and excessive consumption of alcohol. Liver problems can be also caused by infections, autoimmune disorders, and food. This study aims to establish evidence of the use of *Moringa oleifera* in sub-Saharan African countries to manage liver damage conditions in animals.

**Methods:**

In vivo studies will include those in which the activity of the serum levels of hepatic enzymes alanine transaminase (ALT) or serum glutamate-pyruvate transaminase (SGPT), aspartate transaminase (AST) or serum glutamic oxaloacetic transaminase (SGOT), gamma-glutamyltransferase (GGT), alkaline phosphatase (ALP), superoxide dismutase (SOD), catalase (CAT), glutathione reductase (GR), and malondialdehyde (MDA) were measured after administering substances that induced liver injury as a primary outcome.

The secondary outcome will include studies that measure the serum levels of hepatic enzymes (ALT, AST, GGT and ALP, SOD, CAT, and GR), free radical formation represented by MDA after administering the sub-Saharan *Moringa oleifera*, and decreases in their levels indicating the improvement of their activity. Search engines will include MEDLINE, CINAHL, PubMed, Google Scholar, SABINET, EBSCO, and WHO/African Index Medicus. The screened results will be grouped according to any noteworthy grouping variable, such as study characteristics.

Data will be analyzed using Stata statistical software (Stata Corp V.14, TX, USA).

Study data will be quantitatively synthesized by first assessing heterogeneity to examine whether the estimates from included studies could be pooled. Heterogeneity will be assessed by the chi-squared test on Cochran’s *Q* statistic, which will be quantified by *I*^2^ values.

**Discussion:**

Results from this protocol will give new insights into the *Moringa oleifera* plant for developing effective hepatoprotective drugs against liver damage.

**Systematic review registration:**

PROSPERO CRD42018084698.

**Electronic supplementary material:**

The online version of this article (10.1186/s13643-019-1117-2) contains supplementary material, which is available to authorized users.

## Background

One of the most challenging health problems is liver disease, which can be caused by medications, toxic substances, and excessive consumption of alcohol. Liver problems can be also caused by infections, autoimmune disorders, and food. This study aims to establish evidence of the use of *Moringa oleifera* in sub-Saharan African countries to manage liver damage conditions in animals. The liver plays a major role in regulating many of the body’s physiological processes such as its metabolic, vascular, immunological, storing, secretory, and excretory functions. Furthermore, it is involved in detoxifying a variety of drugs and xenobiotics and plays a key role in metabolizing carbohydrate, protein, and fat in the human body [1]. However, the ability of the liver to achieve these functions is often inhibited by many substances which may cause liver diseases. Liver diseases are some of the most challenging health problems, which can be caused by the undesirable effects of some medications, toxic substances, excessive consumption of alcohol, infections, and autoimmune disorders [2]. Liver diseases, which can cause death include cirrhosis, liver cancer, viral hepatitis (B, C, and D), non-alcoholic fatty liver diseases (NALD), alcoholic liver disease, and autoimmune and drug-induced liver diseases [3]. The prevalence of non-alcoholic fatty liver disease was estimated at 20% worldwide in the general population in the year 2012 [4]. Two billion people were infected by hepatitis B worldwide in 2015, and 520,000 deaths were reported, with 170 million being affected by hepatitis C [5]. In India, almost 20,000 deaths are reportedly due to liver diseases every year [6]. Europe reported 63,500 cases of liver cancer in the year 2012, with 29 million people suffering from liver diseases in 2013. In the USA, 30 million people suffered from liver diseases versus 2013, with 170,000 deaths being reported due to liver cirrhosis [5].

Alanine transaminase (ALT), aspartate transaminase (AST), and gamma-glutamyltransferase (GGT) are the serum of hepatocellular enzymes that are normally found in the liver. ALT is the specific marker for liver injury, AST can provide information about other tissues including the heart and skeletal, while GGT is found in the kidney and pancreas [7]. The elevated levels of these enzymes in the serum are a sign of their increased entrance in serum from damaged liver cells [6], with injury being identified using enzymes as hepatic markers [8]. Studies have shown that the chances of the damaged liver being able to heal are reduced in the acute phase which often leads to chronic diseases with complications [9]. In spite of considerable progress in modern medicine, there are very few therapeutic agents that can protect the liver from damage and stimulate liver functions [10]. Patients with chronic liver disease are liable to have liver transplantation, which is not only costly but has long-term consequences of immunosuppressive agents, especially hyperlipidemia, hypertension, and renal disease [11]. For these reasons, many patients with liver disease use herbal remedies to solve their health problems [12].

*Moringa oleifera* is cultivated worldwide, but is a native of India, Bangladesh, and Pakistan [13], being a wild and domestic tree in many sub-Sahara African countries. Phytochemical analyses have shown that its leaves contain various minerals, such as calcium, iron, phosphorus, potassium, and vitamins (A, C, and D) [14]. Its bark, flowers, fruits, leaves, pods, and roots possess many biological and pharmacological properties, such as antibacterial, antidiabetic, anti-inflammatory, antifungal, antihypertensive, antioxidant, antipyretic, antispasmodic, antitumor, antiulcer, cholesterol lowering, diuretic, and hepatoprotective activities in rats [9]. The leaf extracts reportedly reduce serum intracellular enzymes levels by stabilizing the cell membrane, which prevents enzymes from leakages in rats [15]. While the agro-climatic conditions of vegetable materials may vary from one place to another, little is known about the different species of *Moringa* in sub-Saharan countries. This study, therefore, aims to establish the evidence of *Moringa oleifera* from sub-Saharan Africa being used to manage liver damage, specifically in animals. The results will give scientists new insights into the possible use of this plant to develop effective hepatoprotective drugs against liver damage.

### Research questions

For the purpose of this study, the general research question is as follows: Does *Moringa oleifera* from sub-Saharan African manage liver damage? To answer this general question, specific research questions have been developed:

1. Does the administration of substances inducing liver damage increase the levels of hepatic enzyme activities (ALT, AST, GGT or ALP, SOD, CAT, GR, and MDA)?
2. Does *Moringa oleifera* from sub-Saharan Africa decrease the activity of hepatic enzymatic markers?

The study objectives are:

1. To establish whether substances inducing liver damage increase the levels of hepatic enzyme activities (ALT, AST, GGT or ALP, SOD, CAT, and GR) and MDA.
2. To determine whether *Moringa oleifera* decrease the activity of the hepatic enzymatic markers.

## Methods/design

This systematic review is reported following the Preferred Reporting Items for Systematic Reviews and Meta-analyses Protocols (PRISMA-P) 2015 checklist (Additional file 1) [16]. This review protocol having been registered in the international prospective register of a systematic review (PROSPERO), register number CRD42018084698.

### Population

Studies using rats and mice will be selected. Also, studies using *Moringa oleifera* growing in sub-Sahara African countries will be selected.

### Intervention

The intervention will include studies in which administration of substance was done to induce liver damage in rats and mice. The intervention group will include also studies in which *Moringa oleifera* from sub-Saharan African was administered to treat the induced liver damage.

### Comparison

The comparison will include studies in which groups of animals that have and have not been treated with *Moringa oleifera* from within African were compared. This comparison will be evaluated according to the level of liver markers such as ALT, AST, GGT or ALP, SOD, CAT, GR, and MDA.

### Types of outcomes

#### Primary outcome

In vivo studies will include those in which the activity of the serum levels of hepatic enzymes alanine transaminase (ALT) or serum glutamate-pyruvate transaminase (SGPT), aspartate transaminase (AST) or serum glutamic oxaloacetic transaminase (SGOT), gamma-glutamyltransferase (GGT), and alkaline phosphatase (ALP). In addition, studies measuring the primary outcomes and oxidative cascade in serum including the natural antioxidants [superoxide dismutase (SOD), catalase (CAT) and glutathione reductase (GR)] and the free radical formation [malondialdehyde (MDA)] will be included.

#### Secondary outcome

The secondary outcome will consist of studies in which the levels of hepatic enzymes activities of ALT, AST, GGT or ALP, SOD, CAT, GR, and MDA in serum after administration of sub-Saharan African *Moringa oleifera* were measured. This can have positive, negative, or no effects when compared to standard treatment before and after the intervention. Decreases in the levels of those hepatic enzymes in the serum would indicate improvement of their activity after the administration of *Moringa oleifera* (positive effect). Increases in the levels of those hepatic enzymes in the serum would indicate no improvement in their activity after the administration of *Moringa oleifera* (negative effect). Lastly, when there is no change of hepatic enzymes levels when compared to standard treatment before, the case effects will be regarded as static.

### Study eligibility criteria

#### Inclusion criteria

Studies will be included in the systematic review if they satisfied all of the following:

- Research published from 2009 to 2019.
- In vivo research on rats and mice treated with sub-Saharan African *Moringa oleifera* to manage the liver damage.
- Where AST, ALT, GGT, ALP, SOD, CAT, GR, and MDA are measured in the animals’ serum.
- Peer-reviewed literature and non-peer reviewed published articles including scientific reports related to the research topic.
- Case-control groups of treatment with and without *Moringa oleifera.*

#### Exclusion criteria

- Articles on human or animal study other than rats and mice.
- All articles without available full text.
- Articles not addressing the hepatoprotective activity of *Moringa oleifera.*
- Articles using co-administration with another plant.
- Articles using *Moringa oleifera* not harvested in sub-Saharan Africa.
- Case-control group of treatment with another plant than *Moringa oleifera.*

### Information sources and search strategy for identifying relevant studies

A comprehensive and exhaustive search of CINAHL, Google Scholar, MEDLINE, PubMed SABINET, EBSCO, and WHO/African Index Medicus will be conducted. The database will be performed to identify all relevant articles published on hepatoprotective properties of *Moringa oleifera* from sub-Saharan African countries before from 2009 to 2019, regardless of the language of publication. Peer-reviewed articles and subject-related grey literature sources will be part of our search strategy to identify eligible studies. A search strategy based on the combination of relevant terms will be conceived and applied. The following terms and their variants will be used for *Moringa oleifera*: “*Moringa oleifera*,” “Morungue,” “Drumstick tree,” “Horseradish tree,” “Ben tree,” “Moringueiro,” and “Rawag.” For hepatoprotective, we will use the terms “Alanine Transaminase,” “Alanine Amino Transferase,” “serum glutamate-pyruvate transaminase,” “Aspartate Transaminase,” “Aspartate Amino Transferase,” “Serum Glutamic oxaloacetic transaminase,” “Gama-Glutamyltransferase,” “Alkaline phosphatase,” “superoxide,” “dismutase, catalase,” “glutathione reduced,” and “malondialdehyde.” Individual country names for the 46 sub-Saharan African countries will also be used as additional key search terms for more abstracts on the subject. African country names will be introduced both in English and languages relevant to each country for example, “Ivory Coast” and “Côte d’Ivoire.” Where country names have changed over time, old and new names will be included, such as “Zaire” and “Democratic Republic of Congo.” Abstracts of all eligible papers will be reviewed and full texts of articles will be accessed through CINAHL, Google Scholar, MEDLINE, PubMed, SABINET, EBSCO, and WHO/African Index Medicus. The main search strategy conducted in PubMed is shown in Additional file 3. This search strategy will be adapted for searching for another database.

A manual search which consists of scanning the reference lists of eligible papers and other relevant review articles will be also conducted.

### Selection of studies

All studies identified using selected keywords will be screened after removing duplicated articles. The first choice of inclusion criteria for this study will be based on titles and thereafter on abstracts if the title does not clearly give the information related to our study. Full articles will be found for detailed appreciation against the inclusion criteria before screened of articles. When the full text is not available, we will contact authors to request a copy of the paper or we will ask the library to order it. Finally, included articles for the study will be selected in using the above eligibility criteria. Assessment of studies for possible inclusion will be done by two reviewers, and any changes of views will be fixed through discussion until a compromise is reached. Any papers that are not unanimously excluded or included by both reviewers will be examined by both reviewers until an outcome is agreed. If necessary, a third person may be consulted. This will reduce the risk of bias. The review study selection process will be followed by a flow diagram showing the number of all excluded studies as well as reasons for their exclusion (Additional file 2) [17].

### Data extraction and management

A data extraction form will be created by the principal author (WTM) in Microsoft Excel and modified by feedback from three independent reviewers (MGK, MN, and PMOO) to ensure that complete data is obtained. Two review authors (WTM and MGK) will independently extract data. Data will be collected on the first author name; animal species (rats, mice), gender (male and/or female), age (mature adult, middle-aged, old adult), and weight; nature of acute liver damage (drug, toxic chemical, alcohol, food, and infection or autoimmune induced damage); part of Moringa used (bark, flour, leaves, seed), African country of harvest, dose of Moringa administrated, and duration of treatment; and primary and secondary outcome measurements.

Disagreements between authors will be resolved through discussion and consensus, or arbitration by a third author whenever necessary. For managing missing data, we will contact the corresponding author of the respective studies in order to obtain the required details. If no correspondence is received, the study will be included in the systematic review and discussed in the narrative summary.

### Data analysis including assessment of heterogeneity

Data will be analyzed using Stata statistical software (Stata Corp V.14, TX, USA).

Study data will be quantitatively synthesized by first assessing heterogeneity to examine whether the estimates from included studies could be pooled. Heterogeneity will be assessed by the chi-squared test on Cochran’s *Q* statistic, which will be quantified by *I*^2^ values. Values of 25, 50, and 75% for *I*^2^ will be represented low, medium, and high heterogeneity, respectively [18]. When substantial heterogeneity will be detected, we will perform meta-regression and subgroup analyses to investigate the possible sources of heterogeneity using the following grouping variables: animal species (rat , mice), age (mature adult, middle-aged, old adult), gender (male, female, combination of genders used), and weight; nature of acute liver damage (drug, toxic chemical, alcohol, food, and infection or autoimmune induced damage); part of Moringa used (Bark, flour, leaves, seed), African country of harvested, dose of Moringa administrated, and duration of treatment; and outcome measurement results. The heterogeneity between subgroups will be detected by using the *χ*^2^ test on Cochrane’s *Q* statistic. In cases where quantitative synthesis is not appropriate because it is not possible to conduct a meta-analysis due to the heterogeneity of estimates reported by studies, a qualitative narrative synthesis of the evidence will be performed. This will summarize the studies characteristics, animal’s features, nature of liver damage, Moringa features, and outcome result.

### Assessment of risk of bias

The study quality and the presence of potential bias within individual studies will be done at both the outcome level and the study level. Two reviewers (WTM and MGK) will complete the assessment independently. The Systematic Review Centre for Laboratory Animal Experimentation (SYRCLE) risk of bias tool will be used to assess publication bias, addressing assessments for random sequence generation, concealment of allocation, blinding, the outcome measurements, and completeness of outcome reporting [19]. Each bias criterion will be assigned a value of the low, high, or unclear risk of bias for each included study. Quality of studies will be assessed using the CAMARADES checklist [20].

Visual assessment of the funnel plot and the Egger’s statistic will be used to assess for both the presence and statistical significance of publication bias across studies [21].

### Assessment of methodological and evidence qualities

We will use the Grading of Recommendations Assessment, Development, and Evaluation (GRADE) methodology to assess the quality of evidence for each outcome as recommended in the Cochrane Handbook for Systematic Reviews of Interventions [22]. Quality rating of overall evidence will be downgraded according to the following factors: risk of bias, indirectness, the inconsistency of result, imprecision, and publication bias [23]. In addition and where appropriate, the reasons to upgrade the evidence quality will include a large magnitude of effects, a dose-response gradient, and plausible residual confounding that would reduce a demonstrated effect or suggest a spurious effect when results show no effect. We will integrate downgrading and upgrading factors to obtain an overall quality of evidence for each outcome of interest. The overall quality of evidence will be then ranked as high, moderate, low, or very low as specified by the GRADE approach [23, 24].

## Discussion

*Moringa oleifera* is a plant traditionally used for its medicinal and nutritional properties in Africa. Many studies have been done on *Moringa oleifera*, and some of them showed that *Moringa oleifera* possesses hepatoprotective activity [1]. But then, not many studies have focused on *Moringa oleifera* from sub-Saharan countries in managing acute liver damage induced. This protocol states the plan for a systematic review and meta-synthesis of the effectiveness of *Moringa oleifera* from sub-Saharan African countries to manage liver damage. Therefore, this review will fill this gap by revealing evidence that *Moringa oleifera* from different countries of sub-Saharan Africa have the same effects on induced acute liver damage so agro-climatic conditions of vegetable materials may vary from one place to another [25]. Results will be shared with the scientific community through publishing in a peer-reviewed journal. The findings could give scientists new insight into the possible use of this plant to develop effective hepatoprotective drugs that treat liver damage and can serve as valuable inputs for future research in this area.

## Additional files

Additional file 1: Preferred Reporting Items for Systematic Reviews and Meta-analyses Protocols (PRISMA-P) 2015 checklist (DOCX 21 kb)

Additional file 2: PRISMA-P flow-chart of study selection procedure (DOCX 41 kb)

Additional file 3: Search strategy in PubMed (DOCX 15 kb)

## Data Availability

All data generated or analyzed during this study will be included in the published systematic review.

## Abbreviations

ALP: Alkaline phosphatase
ALT: Alanine transaminase
AST: Aspartate transaminase
CAT: Catalase
GGT: Gamma-glutamyltransferase
GR: Glutathione reductase
MDA: Malondialdehyde
NALD: Non-alcoholic fatty liver diseases
PRISMA: Preferred Reporting Items for Systematic Review and Meta-analyses
PRISMA-P: Preferred Reporting Items for Systematic Review and Meta-Analysis Protocol
SGOT: Serum glutamic oxaloacetic transaminase
SGPT: serum glutamate-pyruvate transaminase
SOD: Superoxide dismutase
WHO: World Health Organization
