# Supplementary material for: Mapping the evidence of hepatoprotective properties of Moringa oleifera from sub-Saharan African countries: a systematic review protocol
Source: Syst Rev. 2019 Aug 8;8:197. doi: 10.1186/s13643-019-1117-2 (PMC6688223; doi:10.1186/s13643-019-1117-2)
Supplement: Supplementary file 2 — PRISMA-P flow-chart of study selection procedure (DOCX 41 kb) [file 13643_2019_1117_MOESM2_ESM.docx]

**Additional File 2**: PRISMA-P flow-chart of study selection procedure.

**Records identified through database searching (n=)**

**Additional records identified through other sources (n=)**

Identification

**Records after duplicates removed (n=)**

Screening

**Records excluded, with reasons (n=)**

**Records screened on title and abstract (n=)**

Eligibility

**Full-text articles excluded, with reasons (n=)**

**Full-text articles assessed for eligibility (n=)**

Included

**Studies included in qualitative synthesis (n=)**

**Figure 1**
