# Supplementary material for: Mapping the evidence of hepatoprotective properties of Moringa oleifera from sub-Saharan African countries: a systematic review protocol
Source: Syst Rev. 2019 Aug 8;8:197. doi: 10.1186/s13643-019-1117-2 (PMC6688223; doi:10.1186/s13643-019-1117-2)
Supplement: Supplementary file 3 — Search strategy in PubMed (DOCX 15 kb) [file 13643_2019_1117_MOESM3_ESM.docx]

**Additional file 3 (Table 1): Search strategy in PubMed**

| **Search** | **Area** | **Search terms** |
| --- | --- | --- |
| **#1** | Moringa | Moringa oleifera OR Morungue OR Drumstick tree OR Horseradish tree OR Ben tree OR Moringueiro OR Rawag |
| **#2** | Hepatoprotective | Alanine Transaminase OR Alanine Amino Transferase OR serum glutamate-pyruvate transaminase OR Aspartate Transaminase OR Aspartate Amino Transferase OR Serum Glutamic oxaloacetic transaminase OR Gama-Glutamyltransferase OR Alkaline phosphatase OR superoxide dismutase OR catalase OR glutathione reduced OR malondialdehyde OR hepatotoxicity. |
| **#3** | Sub-Saharan Africa | \| (Angola OR Benin OR Botswana OR Burkina Faso OR Burundi OR Cameroon OR Canary Islands OR Cape Verde OR Central African Republic OR Chad OR Comoros OR Congo OR Democratic Republic of Congo OR Equatorial Guinea OR Eritrea OR Ethiopia OR Gabon OR Gambia OR Ghana OR Guinea OR Guinea Bissau OR Ivory Coast OR Cote d'Ivoire OR Jamahiriya OR Kenya OR Lesotho OR Liberia OR Madagascar OR Malawi OR Mali OR Mauritania OR Mauritius OR Mayotte OR Mozambique OR Namibia OR Niger OR Nigeria OR Rwanda OR Sao Tome OR Senegal OR Seychelles OR Sierra Leone OR South Africa OR St Helena OR Swaziland OR Tanzania OR Togo OR Uganda OR Western Sahara OR Zaire OR Zambia OR Zimbabwe OR sub Saharan Africa). \| \| --- \| |
| **#4** |  | #1 AND #2 AND #3 |
